# Ready or not? Observations from a long-standing community engagement advisory board about investigator competencies for community-engaged research

**DOI:** 10.1017/cts.2018.21

**Published:** 2018-09-03

**Authors:** Alicia K. Matthews, Amparo Castillo, Emily Anderson, Marilyn Willis, Wendy Choure, Kevin Rak, Raymond Ruiz

**Affiliations:** 1 College of Nursing, University of Illinois at Chicago, Chicago, IL, USA; 2 Jane Adams School of Social Work, University of Illinois at Chicago, Chicago, IL, USA; 3 Stritch School of Medicine, Loyola University Chicago, Maywood, IL, USA; 4 The Center for Clinical and Translational Sciences, College of Medicine, University of Illinois at Chicago, Chicago, IL, USA

**Keywords:** Investigator training, community engagement, community engagement advisory boards, clinical and translational sciences, qualitative research

## Abstract

Preparing investigators to competently conduct community-engaged research is critical to achieving Clinical and Translational Science Award (CTSA) program goals. The purpose of this study is to describe the perspectives of members of a long-standing community engagement advisory board (CEAB) on investigators’ readiness to engage communities and indicators of investigator competence in community-engaged research, in order to suggest core competencies to guide the development of CTSA-sponsored educational programs. Two 90-minute focus groups were conducted with a subset of members of a CEAB (n=19) affiliated with the Center for Clinical and Translational Science at the University of Illinois at Chicago. CEAB members identified a range of investigator skills and practices that demonstrate readiness to engage in community-engaged research. Eight competencies were identified that should be incorporated in providing education to enhance the readiness and competency of CTSA-affiliated researchers planning to engage communities in research. CEAB observations demonstrate the necessity of developing competency-based educational programs that prepare clinical and translational scientists at all levels for the important work of community-engaged research.

## Introduction

The National Institutes of Health (NIH) Clinical and Translational Science Awards (CTSAs) program aims to improve the translational research process and maximize the public health impact of scientific discovery [1]. In order to achieve this goal, a specialized translational science workforce is needed [2]. The Institute of Medicine [3] identified the need to create a clinical research workforce adept at the dual tasks of conducting rigorous clinical trial studies related to increasingly complex research questions and translating these results to the community.

In order to provide leadership in workforce development, the CTSA Consortium for Enhancing Clinical Research Professionals’ Training and Qualifications (ECRPTQ) conducted a systematic review of training approaches to establish core competencies across the clinical research continuum [4]. The CTSA ECRPTQ working group adopted the core competency framework established by the Joint Task Force for Clinic Trial Competency which includes 8 thematic areas that are considered critical for early career investigators: scientific concepts and research design, ethical and participant safety considerations, medicines development and regulation, clinical trial operations, study management, data management, leadership, and teamwork [5]. A second work group of the ECRPTQ focused on defining competencies and development of best practices for investigators and clinical coordinators who conduct social and behavioral research [6]. Notably absent in the ECRPTQ recommendations are competencies related to community engagement. Articulation of competencies for community engagement is critical given that the CTSA program encourages and supports community engagement by investigators with limited prior experience or education [7–10]. In addition, many of these investigators are embarking on research projects focused on target populations with whom they have had no prior relationships or perhaps even exposure, further underscoring the importance of establishing core competencies.

Researchers conducting community-engaged research require education not typically offered in traditional graduate or clinical research training programs [11]. Despite the importance of community engagement [12], little has been published describing the goals and priorities of workforce development as it relates to preparation and guidance for successful community engagement. Investigators should not be expected or encouraged—or perhaps even allowed—to engage communities without adequate preparation, which includes education. Instances of ill-prepared investigators causing harm are well-documented; consequences include to the banishment of researchers from certain communities for extended periods of time [13]. Educational programs are needed, and following the ECRPTQ model, these should be competency-driven. Our research aims to contribute to the development of such competencies to fill this gap.

The purpose of this study is to describe the perspectives of members of a long-standing community engagement advisory board (CEAB) on investigators’ readiness to engage communities and indicators of investigator competence in community-engaged research. From this, suggestions will be made regarding core competencies for CTSA-sponsored education for community-engaged research. This information has implications for the development of competency-based education for investigators at all levels as well as study coordinators and staff.

## Methods

### Overview of the CEAB and Relationship to Center for Clinical and Translational Science (CCTS) Activities

With the emerging emphasis on enhancing the translational impact of scientific research, several models of community engagement have emerged with relevance for CTSA awardees. Community advisory boards are a well-described approach for linking researchers to community members and stakeholders and providing consultation on research programs and initiatives [14]. In 2009, the CCTS at the University of Illinois at Chicago (UIC) established the CEAB, a working group of the Recruitment, Retention and Community Engagement Program. Capacity building for academic and community partners in community engagement and translational research is an expressed purpose of the board. The CEAB also includes diverse expertise on the board (lay community members, leaders of community organizations, research staff, and researchers) (for a full description of the CEAB, see [15, 16]).

The chief purpose of the CCTS CEAB is to advise UIC researchers on aspects of community engagement, recruitment, and retention. Consultations are sought by investigators experienced with community-engaged research as well as clinical investigators with limited or no engagement experience. Consultations focus on research methods, recruitment, and retention plans, using culturally appropriate engagement strategies, and identifying and overcoming barriers to participant engagement. The vast majority of consultations have supported an investigator in either implementing a grant-funded study or in preparation for a future submission. There are 2 standing CEAB groups with ~15 members each. The 2 CEAB boards meet on alternating months in order to reduce the overall burden on members. The majority of members serve a 3-year term [15, 16].

#### Procedures

All current CEAB members (n=31) were invited to participate in the focus group sessions. Interested participants contacted the project staff by telephone and were scheduled for a one of 2 focus group dates. The focus group sessions were held at a university location. A total of 19 of the eligible 31 CEAB members participated in the focus group interviews. After completion of informed consent, participants completed a brief demographic survey and were guided through the focus group process, which was conducted according to established focus group methodology [17, 18]. These methods include using a trained moderator (A.K.M.) to guide the structured discussion, the presence of a trained note-taker, established techniques for establishing rapport and group interactions, audio-recording of interviews and immediate post-session facilitator debriefing to highlight important findings, and careful review of professionally transcribed audiotapes. The moderator’s guide focused on a range of topics related to CEAB members’ roles and experiences. Here we report the members’ perspectives on investigators’ readiness to engage communities and indicators of investigator competence to conduct community-engaged research. Focus group participants received a $50 gift card. The study was approved by the Institutional Review Board of the UIC.

#### Data Analysis

Descriptive statistics were used to summarize demographic data. Focus group sessions were professionally transcribed. Two raters reviewed the transcripts for key themes across groups. While keeping the original focus group discussion questions in mind, statements were categorized into broad themes and subthemes. Coding categories were then used to summarize key ideas in the combined focus groups as described by Stewart *et al*. [18]. To protect confidentiality, all recordings, transcripts, and other research documents were logged using participant numbers. Electronic data were password protected. The list of codes linking participant numbers to individuals was stored in a locked filing cabinet separately from all other research documents.

## Results

### Participants

A total of 19 eligible CEAB members participated in the focus group interviews (61%). Participants were primarily female (70%), African American (71%), and were associated with a community-based organization (61%). The majority of participants had been a member of the CEAB for 1–3 years. A few members had served multiple 3-year terms. Key qualitative findings are described in the next section and organized based on broad themes and subthemes. Quotes illustrate main points.

### Perceptions of Investigators’ Readiness to Engage Communities in Research

Focus group participants were asked to reflect on the research consultations that they have participated in as members of the CCTS CEAB consultation service. Specifically, they were asked to discuss their initial impressions of the readiness for community engagement demonstrated by researchers seeking consultation and to reflect on their “gut reactions” regarding those investigators they perceived to be less prepared and potentially more at risk for unsuccessful community engagement attempts.

Members acknowledged a wide variability in training and prior experience among researchers. All consulting investigators were perceived as working on projects with potential benefit for improving the health of community members. Although the majority of investigators who have presented before the CEAB have had some prior experience with community engagement, some investigators were clearly memorable, and often junior investigators seeking consultations have had very little formal training or exposure to either the science of community engagement [19] or its practical application. One member stated:

*“I’ll say, very respectfully, sometimes I’ve seen the expressions on some of the [faces of] investigators [are] like deer in headlights. I really have. I say that in all due respect.”*

Another said, “They don’t understand some of the things [about community engagement] that we take for granted.”

Other investigators were seen as highly prepared and capable of conducting highly impactful research projects. As one member observed:

*“When I walk out of here some days, I’m thinking like, ‘Man, they’re really lost in terms of what they’re trying to do.’ Then I walk out of some meetings. They’re right there. They’re almost there. They’re doing some really good stuff. It’s [the project] going to be impactful.”*

Many of the consultations conducted were with investigators and research teams experiencing difficulties in participant recruitment. Typically, in these situations, the principal investigator had written a grant application, obtained funding, and finalized the research protocol without first engaging communities to ensure relevance, buy-in, partnership and cooperation of community members in achieving study objectives—most critically, individual participant participation. Describing one such consultation, a CEAB member said:

*“There’s no engagement aspect to it. There’s no population—none of the population has been invited to the table, even in the formative stages. There’s been no discussion with anybody from that particular population. They don’t have a sense of what area to target, or what community-based organizations, or who the stakeholders are. Those are, to me, signs that you don’t really understand who it is you’re trying to engage.”*

Another typical reason for a consultation is an investigator who is aware of the importance of engaging community members and seeks assistance in developing community relationships. In these situations, board members felt investigators often lacked an appreciation of the time and process required to build these relationships. One CEAB member reflected on their observations of the attitudes of newer investigators regarding relationship building:

*“I just feel like, for the newer investigators that we’ve heard, I don’t hear what the process of relationship building is going to be. I know that it’s not a fast and easy process. It’s actually a journey. It is important to instill [in young investigators] or to emphasize that you have to do that work [of relationship building], if you’re gonna sustain your presence.”*

CEAB members’ observations on investigator preparedness reflected their level of “proximity” to underserved communities. Those CEAB members who represent grassroots community organizations reported the most concerns about less experienced investigators. In these instances, several members expressed concerns about linking investigators that appeared under-prepared to specific community organizations and members. Member hesitancy resulted from their perceived role as “gatekeeper”; they saw themselves as responsible for providing researchers with access to communities, but also responsible for “keeping out” any researchers who do not seem prepared to work with community, whose research does not offer obvious benefits to community members, or who have not established true partnership with community-based organizations.

### Indicators of Investigator Competence for Community-Engaged Research

When asked to think about investigator characteristics, statements, or actions that indicate competence to engage in community-engaged research, CEAB members expressed a range of perspectives.

#### Purpose of Research

For some members, the skills or qualities of the researcher were less important than the focus of the research project itself. For those members, the potential benefit of the study for community members superseded other factors. For example, one member responded:

*“I think for me it’s the purpose of the research. It’s their own goal with the research, what they really wanna accomplish with it.”*

#### Communication

For other CEAB members, a key indicator of an investigator’s competence to appropriately engage in community-engaged research is their demonstrated ability to communicate respect and sensitivity for the issues facing the community they are trying to engage.

*“I think, right off the bat, respect and understanding of the population they’re intending to study. That comes across loudly, I think, when they stand before us and present. Sometimes it just feels like they don’t have any clue about the people that they’re intending to work with. Other times, it feels like they have done their homework. For me, I have to sense that they have a respect and understanding for whoever it is they’re targeting.”*

The ability to effectively communicate with lay community members was seen as a reflection of investigator competence to conduct community-engaged research. The most consistently cited issue associated with communication was the tendency for some investigators to speak to the CEAB group in technical language or discipline-specific jargon. As some members said:

*“There are people who are very technical, and there’s no relationship conversation. There’s no discussion of relating to the group.”*

*“You have to throw some of that book stuff out the window and talk for real to the community.”*

However, other members felt that use of technical language was not necessarily a problem as long as appropriate explanations were given, but they were concerned when investigators refer to community members in terms of a disease state as opposed to individuals living with a specific disease (i.e., AIDS patients vs. people living with AIDS). This nuance in language was seen as conveying sensitivity and respect for community members.

Other communication concerns were specifically related to communication about projects either not getting funded or ending due to a lack of funding. Members recounted experiences that they have had of researchers simply disappearing following the completion of a grant. Members strongly recommended that researchers clearly communicate with community stakeholders throughout the course of the grant, set expectations for ending or sustaining relationships following the completion of the study, and provide feedback about the study findings and the community value in the completion of the study.

*“if I were informing or giving feedback to a researcher, going forward, I would perhaps ask a question: what happens if your project doesn’t get funded at the next level? How do you, then, get back to the people who have participated and say sayonara, without just leaving ’em hanging? Say what the value of it has been, but I’m sorry, we can’t continue to work on this, but this is what the value has been so far.”*

#### Openness to Feedback

Some members reflected that the dynamics between the investigator and the CEAB members during the research consultation service offered insight into an investigator’s competence to participate in community-engaged research. In a typical consultation, investigators present to the CEAB board members with specific questions. An investigator’s openness to feedback from CEAB members was viewed as in indicator of how they may interact with community members during research implementation.

*“Depending on their plan and their response, you can either lead them, or you can leave them. It’s up to them. If they wanna be led, you ask the right questions, and you lead them, and they follow.”*

#### Cultural Sensitivity

CEAB members emphasized the importance of cultural competency and sensitivity in demonstrating readiness to engage in community-engaged research. The group acknowledged that not all investigators have had the lived experience, education, or other exposure that would adequately prepare them to interact with particular communities. Central to competence to succeed in community-engaged research is training in engagement across differences related to race/ethnicity, class, gender, sexual orientation, and immigration status, to name just a few dimensions of difference.

However, CEAB members noted certain indicators of cultural sensitivity—or sensitivity to their own limitations—for example, the construction of a diverse research and outreach team that both reflects the demographics of the target population and has an understanding of the socio-cultural and historical experiences of that group. One CEAB member recommended:

*“If you are a totally different ethnic group from the group you want to study, and you have not had any meaningful experience working with that group, you do need to consider having, on your team, people who are also on that group.”*

The goal of cultural competency training is not to just be aware of the superficial characteristics of different communities and their members but to also understand the experiences of those communities with regard to research participation—both historically and with the investigator’s institution. For example, although the specific individual investigator may be well trained in community-engaged research, they may be located in an institution with a mixed reputation within the target community. This reputation may also include the perception of exploitation or lack of true commitment to community engagement. For example, one member described what they perceived as a very one-sided relationship between local research institutions and his community.

*“I think that we give a lot and we don’t get a lot back. They still come back to pretty much get more from us.”*

Similarly, another participant said,

*“We’ve participated in research. We help folks get their numbers together. We help them. We’ve invited and held their hands, bring ’em into the hood, and then they disappear. I’ve been doing that for 15 years.”*

Both background knowledge of and an ability to directly address the history between research institutions and communities is necessary to repair damaged reputations and establish strong, new collaborative partnerships.

#### Community Presence

Regardless of the strength or diversity of their research team, CEAB members also felt that the lead investigator should have an actual presence in the community and not simply send project staff to engage with community-based organizations and community members. The absence of the principal investigator in community engagement activities was perceived by CEAB members as conveying a lack of commitment to the community. As described by CEAB members:

*“For me, just from my experience and what I’ve observed, the most successful researchers or principal investigators are those that are engaged in the community. Meaning, they’re not just sending out their field workers out into the community to collect the data, or to run those programs. Their presence is felt and known in the community.”*

*“It can’t be a presentation one Sunday or one Saturday. It has to be ongoing, with an opportunity for ongoing conversation back and forth, an opportunity for ongoing operational issues to be resolved back and forth.”*

These commitments go beyond what one member referred to as photo-ops:

*“I’ll just tell you the truth. You listen [to the researchers]. Everybody takes their photo-ops. Then, it’s over with.”*

#### Power Sharing

Another foundational skill is the ability to establish equitable partnerships. Power sharing should include shared decision-making, resource allocation, and benefits and costs of the research endeavor.

*“With community-based research, you’re sitting at the table with your potential research partners and there’s gotta be the bidirectional co-learning, give and take, et cetera, et cetera, because we’re all level.”*

Creation of equitable relationships can be contrasted with relationships that are typified by power concentrated within the university partners. CEAB members recounted several personal examples of partnerships where the community partner was viewed only as a recruitment site and not a true partner.

*“With me, as a community-based organization,—they’ll call and they say, ‘Oh, I’m gonna drop off some stuff. Can you pass this out? Can you do this?’ That’s not gonna work. That’s not gonna work.”*

The importance of bidirectional dialog was discussed repeatedly by CEAB members. First, members discussed the importance of maintaining consistency and transparency in communicating with community partners in order to build new relationships and to maintain existing relationships. Members also emphasized the importance of having procedures in place for dealing with problems that may emerge in order to be able to:

*“Address all the things that can occur, as well as complaints, a complaint process, or a problem with the research itself, so that you don’t get this—cuz once you get a reputation for not being responsive, then nobody is going to receive you again.”*

#### Recognizing Partner Contributions

Investigators should also acknowledge and value the contributions of community partners. Community partners should be acknowledged for the intellectual contributions to the scientific process as well as the connections they can provide. One member summarized the issue in this way:

*“Just respect and value your community partners. Understand the human capital, the social networks, is the intellectual property [of the community] that comes out of your questioning. I mean, if you don’t have that then you don’t have a research project.”*

#### Developing Community Capacity

Capacity building is one mechanism for maintaining stakeholder engagement during and after the active phase of the research project. Training of community members to continue to deliver an intervention after the completion of the study is one example of capacity building.

*“Part of that component is bringing back the community partners, and really working with them, and outlining, okay, how can we make sure that not only are we getting the results and doing the program that we want, but that we’re training our partners or key members of that community to help us to continue on, once we leave.”*

## Discussion

During the past decade, there has been a growing interest in learning and competency-based systems in various areas of education, training, and professional development associated with research [4]. The purpose of this study is to describe the perspectives of members of a long-standing CEAB on the readiness of and desirable competencies for investigators in the conduct of community-engaged research. Based on the 8 themes that emerged from the focus groups with CEAB members, we have developed a list of preliminary competencies for community engagement to guide the development of training and continuing education for CTSA-affiliated investigators and research coordinators (see Table 1). Many of the suggested foci for training are consistent with principles of community-engaged research [11, 20, 21], while others have not been described as part of the core competencies related to community-engaged research.

**Table 1:** Using a Knowledge, Skills and Attitudes Framework to propose educational competencies for community-engaged research

Knowledge
1. Articulate a community-centered focus and benefit of the research project and goals
2. Demonstrate respect and sensitivity for the issues facing the community one wants to engage with by effective communication and knowledge of the socio-political history of the group in the United States and locally
Skills
3. Demonstrate openness to community input, perspectives, and priorities by establishing a collaborative team-based approach
4. Demonstrate cultural sensitivity, competency, and ability to engage across differences related to race/ethnicity, class, gender, sexual orientation, and immigration status, to name just a few
Attitudes
5. Appreciate the importance of maintaining a presence and leadership in community engagement activities with community-based organizations and community members
6. Value equitable partnerships exemplified by equal power sharing, decision-making, resource allocation, and costs associated with the research
7. Acknowledge and value community partner intellectual contributions to the scientific process
8. Value community capacity building as part of the key research outcomes and deliverables

The Centers for Disease Control’s Knowledge, Skills and Attitudes competency model represents a useful heuristic for developing and organizing the emerging competencies for CTSA researchers in the conduct of community-engaged research [22]. Knowledge is the first of 3 global domains for competency in the Knowledge, Skills and Attitudes Framework and refers to the complex process of remembering, relating, or judging an issue or problem area. As in other areas of education and training [21], competencies in community-engaged research represent not only basic knowledge, but the ability to effectively communicate that knowledge [4]. Board members want clear, jargon-free, culturally sensitive communication that demonstrates respect for the local culture, and appreciation for their contributions. They want the creation of mechanisms for conflict resolution and that keep residents from feeling they have been taken advantage of. The commitment of the researcher is manifested by a genuine interest in the community and their continuous presence before, during, and after the end of the project at hand. The underlying expectation is that the researcher will be prepared to be influenced by the community, will be patient with the process and will work with the community to sustain programs and outcomes. These competencies are reflected in community-engaged research scholarship, but it is critical to have expert community voices contribute to the discussion.

The second global competency domain outlined in the framework is skill. According to the framework, skills are the behaviors (manual, verbal, or cognitive) that allow for the execution of well-specified tasks. As could be expected in the interaction of 2 different perspectives, language, communication and cultural competence are critical skills in a successful collaboration between researchers and community representatives [23–25]. An important concept associated with engagement across difference is cultural competency, or the ability to interact effectively with people of different cultures [26]. However, beyond cultural competence training, there is a call for the merging of purpose, to search and to find mutual interests that benefit both groups, and dissipate distrust and skepticism. This call for effective communication is also found in the work of other authors [23, 24, 27].

Attitude is the final global component in the model and represents a state of mind, feelings, or beliefs about a particular matter. Significant time and emphasis were given to discussing the readiness of the researchers to engage in community work. A lack of preparation is perceived as one of the reasons for insensitive communication and impatience with the research process. CEAB members’ candid revelations parallel some of the observations by Sprague-Martinez *et al*. [24], who describe the request of their community members for clear language, but also for an elevation of the communication so they do not feel “talked down” by the researchers. Their participants also wanted to see long-term relations, and investment of the researcher on shared interests. Their requests, like that of our CEAB members, emphasize the practice of community engagement in a way that is respectful of community and its resources, requires transparency, collaboration in planning and implementation, and projection into the future. These factors define any collaboration as a long-term endeavor for which most researchers are not clearly prepared.

Our participants also expressed their expectation that the researcher’s work should endure and create long-term effects in the life of the community. Lack of attention to sustainability, coupled with limited control over financial resources, engender mistrust and the perception that the community is being taken advantage of. Financial control over resources is considered central to equalizing the relationship with communities [28]. The involvement of community members as part of the research team offers benefits by enhancing the community’s human resources and the cultural relevance of the research practices. The emerging role of the “Ph.D.’s of the sidewalk” [29] or “citizen scientists” [30] is a framework for understanding the value of community members beyond venues for recruiting for research studies. The capacity building involved in the implementation of community-engaged research, strengthens community ownership of resources and facilitates the sustainability of projects beyond the funding cycle [25]. And all this investment in the life of communities, including what represents the researcher’s career, generates long-term binds of trust and mutual support.

Our study has some limitations. First, CEAB members represent a unique group. Some of them are familiar with the research enterprise as former employees of academic organizations, and others are leaders of community organizations. As such they may not always reflect the experience of grassroots members of the community. However, their experience of both community and academic worlds give them a unique and valuable perspective in evaluating researchers’ preparedness and facility for community engagement. Second, the unique history and composition of the board may not allow for the generalizability of their responses to other advisory groups. The observations of our board members, however, seem to reflect similar themes as those reported by other authors [23, 27, 30]. A strength of the CEAB perspective is that consultations are conducted with a broad range of investigators at an institution where community engagement is widely promoted and valued.

## Conclusion

Community engagement is essential to the successful translation of interventions and other healthcare advances into community settings. However, competency-based education is required to increase the preparedness and skills needed for community-engaged research. Educational opportunities can take the form of fellowship programs, courses, conferences, science cafes, certifications, workshops, and service learning approaches [31]. Whatever form or format, educational programs should help investigators at all levels develop core competencies. These should be identified by a robust process like other competency training programs. Input from experienced community partners and community-engaged researchers should guide development of training approaches for CCTS affiliated investigators. Here we have started the conversation. If CTSAs believe in a bright future for translational science, it is necessary to enhance the profile of community engagement practices in the education of students and fellows, and prepare them for the long-term commitment with community that is required for successful partnerships and outcomes. Equally important would be to give recognition to those skills by taking them into account for job promotion.
